# Mercury in the feathers of Golden eagle (*Aquila chrysaetos*) from Western Carpathian, Slovakia

**DOI:** 10.1007/s11356-024-32673-8

**Published:** 2024-03-06

**Authors:** Terézia Sabadková, Marián Janiga, Ján Korňan, Tatiana Pitoňáková

**Affiliations:** 1 Institute of High Mountain Biology, University of Žilina, Tatranská Javorina 7, 059 56 Tatranská Javorina, Slovakia; 2 State Nature Conservancy of the Slovak Republic, Bratislava, Slovakia; 3 University of Veterinary Medicine and Pharmacy in Košice, Komenského 73, 04181 Košice, Slovakia

**Keywords:** *Aquila chrysaetos*, Habitat, Western Carpathian, Mercury, Bioaccumulation, Predatory birds, Monitoring

## Abstract

In this study, mercury (Hg) concentrations were detected in feathers of golden eagle (*Aquila chrysaetos*), a bird that typically inhabits alpine and forest areas. The mercury rates in feathers were compared in two groups of eagles: first, estimated home range (breeding, hunting, etc.) was located only in forest and lowland meadow habitats; second, the home range also included alpine habitats—rocks and meadows. Consequently, mercury concentration based on the feather typology were observed and the mercury levels in feathers were also compared among different Slovak Western Carpathian districts. It was found that there was no significant difference between groups classified by elevation level, which we attribute to the fact that eagle hunting territories are broad, so that alpine-dwelling and forest-dwelling eagles do not only reflect the pollution of the environments they typically inhabit. Non-significant differences were found also within different feather types, which means that the type of feather is not crucial for tracking mercury in eagle feathers. As the measurement of feather appears to be a simple and non-invasive method, the detection of non-significant differences in diverse types of golden eagle feathers provides useful knowledge for the future environment monitoring. The average mercury concentration measured in eagle samples was lower than the mercury concentration causing health complications among birds of prey. Our assumption that due to past mining activity in the Spiš region, the highest concentration in this region would be observed was confirmed.

## Introduction

Atmospheric Hg^0^ has a short half-life in polar regions and is therefore rapidly methylated by microorganisms in the aquatic environment to methylmercury (MeHg). In our research, we work under the assumption that total mercury is primarily composed of methylmercury, as reported by other studies in feather, liver, kidney, and muscle samples (Thompson and Furness 1989; Burger and Gochfeld 1997). This form is the most toxic and reaches the highest levels via the food chain (Driscoll et al. 2013; Vizuete et al. 2019). According to Stankovic and Stankovic (2013), Hg is classified as toxic heavy metal that is easily exposed. Because of their toxicity, bio-accumulative, and non-biodegradable nature, heavy metals are considered potentially hazardous to aquatic, animal, and human life (Balintova et al. 2012; Stankovic and Stankovic 2013). At very high exposure levels, toxicity among animals is associated with disease of the central nervous system, kidney, or stomach damage, and affects reproduction, leading to population decline (Scheuhammer et al. 2007). The highest total Hg concentrations in mammals and birds are in internal organs (liver, kidney, muscle, and brain) (Wagemann et al. 1998; Mieiro et al. 2009). Subsequently, methylmercury is incorporated into growing keratinized tissues such as feathers, claws, hair, and scales (Chételat et al. 2020). Methylmercury thus bound in keratinized tissue represents the dominant elimination pathway for birds (Nichols et al. 2010; Jaspers et al. 2019). These metabolically inert tissues form an inert complex with MeHg during growth, conserving the MeHg bioaccumulation index over time periods (Chételat et al. 2020). That is, metal accumulation in feathers generally represents a longer-term contamination process, whereas blood levels represent recent contamination directly associated with feeding (Carvalho et al. 2013). Feather concentration levels reflect body accumulation throughout the period of feather development (Borghesi et al. 2017). Due to the bioaccumulation of mercury, animals that inhabit the study area can be used as bioindicators of environmental pollution (Gadzala-Kopciuch et al. 2004). Birds are a favoured group for bioindication because of their abundance, distribution, representation of different trophic levels, length of life, and susceptibility to anthropogenic pollution (Edmonds et al. 2010; Townsend et al. 2013; Hartman et al. 2013; Jackson et al. 2016). In this study, feather samples of golden eagle (*Aquila chrysaetos*), which belongs to the group of raptors, were used for monitoring of environmental pollution by mercury. Because birds of prey are often protected, it is necessary to use non-invasive methods of sample collection (Hopkins et al. 2007). Compared to the use of bird tissues, the main advantages of sampling feathers is the simplicity of storage and the high stability of Hg in the feathers, due to the tight binding of Hg to the disulfide bonds of the keratin (Crewther et al. 1965; Movalli 2000).

The golden eagle is not considered endangered, and the IUCN has classified the golden eagle as of least concern on its Red List of Threatened Species (International) 2021). Nevertheless, it is often listed as a cosmopolitan but rare species, which is affected by several factors (Altmeyer et al. 1991; Esselink et al. 1995). In a given context, not only external but also internal factors are also important determinants which influence the amount of mercury in the animal bodies (Pedersen and Lierhagen 2006). Internal factors include mainly nutritional conditions, quality of food, feeding habits, and physiology of the animal body (Ali et al. 2019). In the Western Carpathians, local birds or pairs hunt in the alpine resort, mainly near alpine meadows or lakes. They tend to favor diet rich in Tatra marmot (*Marmota marmota latirostris*) and juvenile chamois (*Tatra chamois*) but can enjoy a far more diverse diet based on their extensive range. They often hunt field hare, martens, weasels, wild and domestic cats, dogs, vipers, young roe deer, and squirrels (Tjernberg 2006).

In Slovakia, the golden eagles occupy breeding habitats in elevational range from 400 to 1700 m above sea level. Eagles often hunt at even higher elevations (up to 2500 m a.s.l.) in the High and Low Tatras regions (Korňan et al. 2023). Alpine areas are considered to be a reservoir of mercury compounds, where toxin pollution is not only caused by the movement of toxins in rivers and intoxication of organisms inhabiting rivers and lakes but also by long-range wind deposition (Shao et al. 2016; Tripathee et al. 2019). The available literature describes that in Europe, the most polluted areas are the highly populated and industrialized parts of Eastern Europe, where long-distance transport of pollutants are also responsible for the formation of metal pollution stress in alpine ecosystems (Šoltés 1992). The issue of heavy metal pollution has been resonating in the Western Carpathians for a long time. A study by Ballová et al. (2020) focused on the relationship between heavy metal accumulation and histological alterations in voles from alpine and forest habitats reveal that heavy metals (Hg and Pb) accumulate to a greater extent in femoral bones of alpine habitats than forests. For other contaminants, such as Pb, an increasing effect of concentration with altitude has been observed (Janiga et al. 2012), though this trend was not observed for Hg (Poláček and Haas 2018).

The first aim of this study is to determine whether any type of eagle feather can be used in environmental mercury monitoring. The second, and in our case more important aim of the study, is to determine the mercury levels in eagle feathers from different regions of the Slovakian Western Carpathians, and to compare mercury concentrations in feathers from eagles from forests and lowlands to samples from eagles from alpine ecosystems. Since we assume that the diet of alpine and forest-dwelling eagles differs, detecting mercury concentrations in the feathers of eagles can help to visualize environmental pollution levels in these two habitats.

## Materials and methods

### Sampling sites and sample collection

Our research area was situated in the Western Carpathian Mountain range of Slovakia. Feather samples were collected under the project entitled “Monitoring and management of the golden eagle (*Aquila chrysaetos*) population in Slovakia” between 1994 and 2002 (Korňan 2003). All birds included in the analysis (*N* = 100) were free-living and naturally feeding. Feather samples were taken directly from nests. For site identification, one feather was selected from each nest during breeding activity from each year. As the different feather types did not differ significantly in mercury content (see “Results and discussion”), it was possible to use different feather types to compare mercury levels among regions or habitats. A total of 129 feathers were collected randomly during monitoring, and subsequently sorted by habitat (i.e., alpine vs forest); historical districts (Liptov, Turiec, Orava, Horná Nitra, Kysuce and Horné Považie, Spiš); and the body part from which they originated (remiges, rectrices, tectrices, plumae breast, plumae rump, plumae undertail, plumae other). A separate group “plumae other” was created for those feathers whose origination could not be identified. The number of N’s in the category of body part from which the feather originated varied because more than one type of feather was available from a single individual. Most feather samples were collected during summer months when molting is the most intense.

### Laboratory analysis

Samples were stored in sterile bags and kept in the fridge (4 °C). From each sample, a piece measuring approximately 1 × 1 cm was cut from the outer space of feather. Samples were cut using stainless steel scissors cleaned with ethanol before and after each use. The exact weight of each sample was determined, using an accuracy of 0.001 g on the balances of a Kern 770 (Kern & Sohn, Germany). The results are presented in dry weight of the sample. The DMA-80 mercury analyzer (DMA – 80 Dual Cell, Milestone srl, Italy) was used to determine the mercury concentration in samples. Nickel boats were used, and they were cleaned after every four measurements; the cleaning process was conducted until stable absorbance was less than 0.003. The following settings were used: combustion temperature of 650 °C, catalyst temperature of 615 °C, and cuvette temperature of 125 °C. The accuracy of the method for the detection of total mercury in feather was achieved by analysis of the reference material Beef liver NCS ZC 71001 (CHNACIS, China). The abovementioned reference material was used to check the quality of the measurements and to validate the measurement method. The limit of detection (LOD) and limit of quantification (LOQ) were determined by 10 independent analyses of a blank (empty sample plate). The equations used for calculation were as follows: LOD = 3 × s; LOQ = 10 × s (s = mean standard deviation, calculated from 10 independent blanks). LOD was set at 0.003 μg/g and LOQ at 0.0094 μg/g. The determined method showed a high average % recovery (> 95%) in the matrix of standard reference material and a relative standard deviation (RSD) less than or equal to 5%.

### Statistical analysis

Statistical analyses were conducted with Statistica ver. 12 software for Windows (Stat Soft CR, Prague, Czech Republic). Only samples containing all necessary information with regard to their origin were used for statistical analysis of each group. Differences in mercury concentrations in feather types were compared using the non-parametric Kruskal–Wallis test. Mercury concentrations were also compared between different habitat (alpine and forest) and between historical districts using the Mann–Whitney *U* test.

## Results and discussion

### Feather types

Average and median mercury concentrations within feather types of golden eagle samples were compared, and the results are shown in Table 1. The table shows that the lowest average mercury concentration was found in the plumae undertail, and the highest in rectrices. Seven types of feathers were sampled. From the highest to lowest mercury concentration according to mean, they are listed as follows: rectrices, plumae breast, tectrices, plumae rump, remiges, plumae other, plumae undertail. Significant relationship between the amount of Hg within eagle feathers was not found (*p* = 0.63).

**Table 1:** Descriptive statistics of T-Hg (mg/kg) depending on feather type, historical districts, and habitat

		N	Mean (mg/kg)	Median (mg/kg)	Min (mg/kg)	Max (mg/kg)	SD (mg/kg)	SE (mg/kg)	*p*	
Feather type	Remiges	19	1.68	0.93	0.21	5.73	1.84	0.42	0.63	KW-H = 4.35
	Rectrices	11	2.31	1.03	0.54	8.08	2.53	0.76		
	Tectrices	36	1.77	1.13	0.24	6.72	1.59	0.26		
	Plumae breast	19	1.91	1.56	0.38	5.82	1.51	0.35		
	Plumae rump	12	1.71	1.30	0.13	4.37	1.50	0.43		
	Plumae undertail	19	1.51	1.36	0.28	3.70	1.11	0.26		
	Plumae other	13	1.53	0.78	0.16	7.63	2.12	0.58		
Historical districts	Liptov	34	1.49	1.04	0.13	5.82	1.32	0.23	0.83	KW-H = 2.15
	Turiec	10	1.97	1.47	0.45	8.08	2.28	0.45		
	Orava	13	1.46	1.08	0.43	4.29	1.03	0.43		
	Horná Nitra	14	1.78	0.92	0.26	6.72	1.80	0.48		
	Horné Považie, Kysuce	19	1.70	1.06	0.44	5.05	1.25	0.29		
	Spiš	10	2.81	2.07	0.23	7.63	2.59	0.82		
Habitat	Forest	48	1.90	1.05	0.13	8.08	1.63	0.27	0.58	MW-U = 1168
	Alpine	52	1.61	1.13	0.16	5.82	1.36	0.19		

**Fig. 1:**
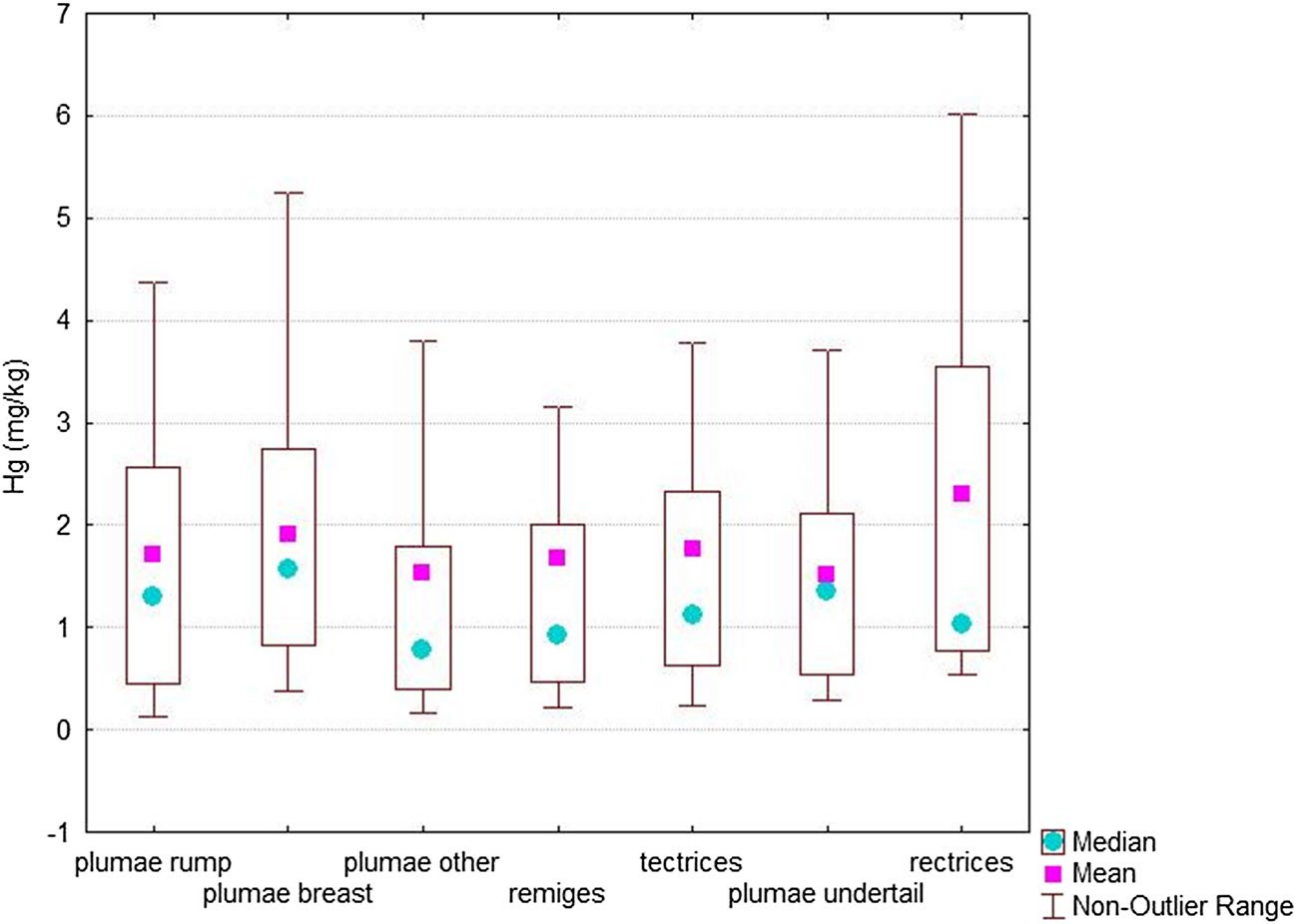
Mean mercury concentrations and median value in different feather types of golden eagle. The groups did not differ significantly (Hg (mg/kg): KW-H (6.129) = 4.3499, *p* = 0.6294)

In most published studies aimed at measuring the concentration of Hg in the feathers, remiges or rectrices were used; usually primary or secondary feathers or middle tail feathers, since they are considered to be representative for the measurement of total mercury in feathers (Furness et al. 1986). However, in our case, when measuring the amount of mercury in individual feather types, we did not find significant differences in mercury levels among types of feathers. Therefore, for the purpose of measuring mercury, we have not considered different types of feathers as representative. Several studies have shown that the amount of Hg varies from one part to another in different individuals (Berg et al. 1966; Goede and de Bruin 1984) and also varies between different parts of the feather (Buhler and Norheim 1981; Doi and Fukuyama 1983; Appelquist et al. 1984). The difference in accumulation in different feathers was also investigated by Low, who concluded that Hg concentrations in the body feathers of songbirds with average concentration 0.50 mg/kg were similar to those in primary feathers with an average concentration of 0.57 mg/kg (Low et al. 2020). The mercury values we found in golden eagle primary feathers (x̄ = 1.94 mg/kg) tended to be higher than those measured in body feathers (x̄ = 1.69 mg/kg), but difference was non-significant. This may be because amount of mercury stored in body tissue is the main factor that determines Hg levels in plumage; molting reduces the concentration of mercury in the feathers and as the primary feathers are usually the first to be molted, the higher variation was measured in these compared to body feathers (Furness et al. 1986). Harmata and Restani (2013) also investigated mercury content in feathers and blood in southwestern Montana, but their results did not exceed concentrations greater than 1 mg/kg in feathers. The concentrations from the West Carpathians were on average higher in all feather types (Fig. 1).

Ginn and Melville (1983) found that mercury levels in rectrices are usually higher than in secondaries, but lower than in primaries. Our results confirmed that measured mercury levels tend to be higher in rectrices than in other feather types. They also report that differences in mercury levels are lowest in body feather samples, and as such, these feathers are suitable for biomonitoring of mercury (Ginn and Melville 1983). Our results also support the suggestion that the smallest differences are in body feathers. However, when looking at the results as a group, we can conclude that the differences in other feather types also did not appear to be significant. From this observation, we can conclude that any type of feather can be used for mercury monitoring of golden eagle species. The importance of this finding is related to the fact that monitoring mercury in randomly dropped feathers is a non-invasive and non-lethal method, compared to body organ analysis; the animal is not under stress and samples can be stored for long periods of time, as mercury is tightly bound in the feathers by a disulfide bridge and stable for many years (Hopkins et al. 2007).

### Health

**Fig. 2:**
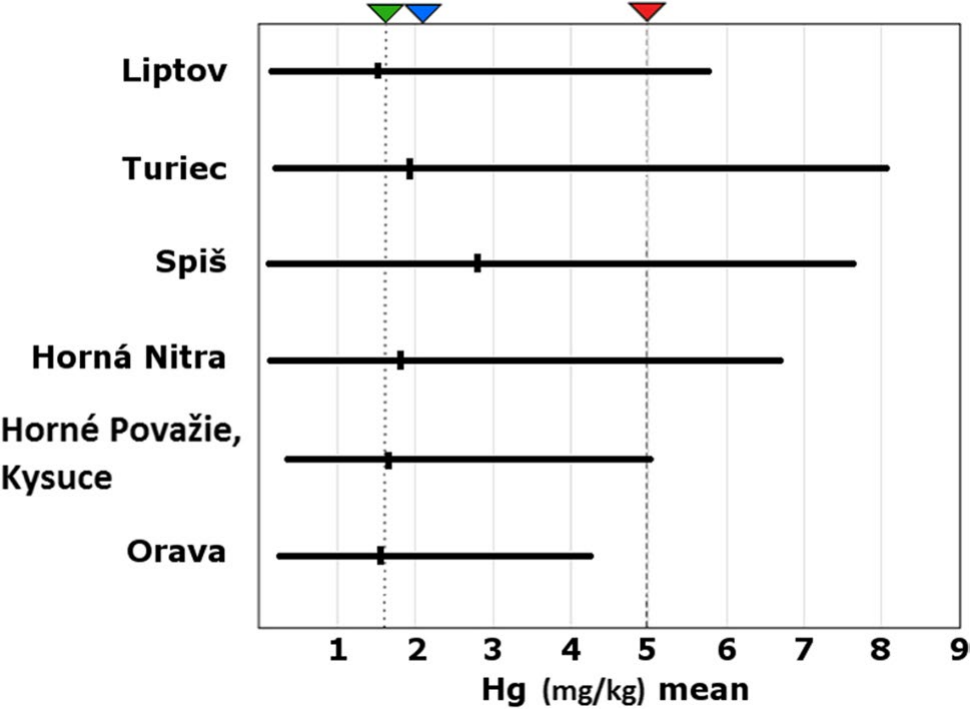
Level of Hg (mg/kg) in the feather of golden eagle from different regions. The horizontal line is the range (min–max); mark on the horizontal line is the mean concentration of Hg for each region. The green triangle and vertical dotted line points to the mean of Hg level in our research. The mean value for the studies of birds of prey reviewed by Burger (1993) is illustrated by blue triangle. Red triangle points to the adverse effect, the limit of which has been established by laboratory studies reviewed by Eisler (1987)

Mercury can enter an animal’s body through food, starting with small rodents (Abt and Bock 1998). The phenomenon of mercury toxicity in small rodents was described in a recent study on snow voles *Chionomys nivalis* (Martinková et al. 2019). For birds of prey, which are high up on the food chain, biomagnification is common, with serious consequence in wildlife (Wolfe et al. 1998; Albers et al. 2007; Heinz et al. 2009; Ackerman et al. 2007). The mean mercury value (1.75 mg/kg) in this study was lower than the calculated mean value (2.1 mg/kg) from 180 studies measuring mercury in bird feathers (Burger 1993). Compared to Wood’s study of raptors, our values are also below the threshold for causing behavioral changes and reducing reproduction (Wood et al. 1996). According to Eisler, values associated with adverse effects range from 5 to 40 mg/kg (dry weight) (Eisler 1987). The average value from our study does not exceed this limit, but when samples are analyzed individually, several values exceed the limit. The highest outlier value recorded was 8.08 mg/kg, which is a value associated with toxic effects on health (Fig. 2). Eleven eagles exceeded the limit of Hg concentration above 5 mg/kg.

### Locality

**Fig. 3:**
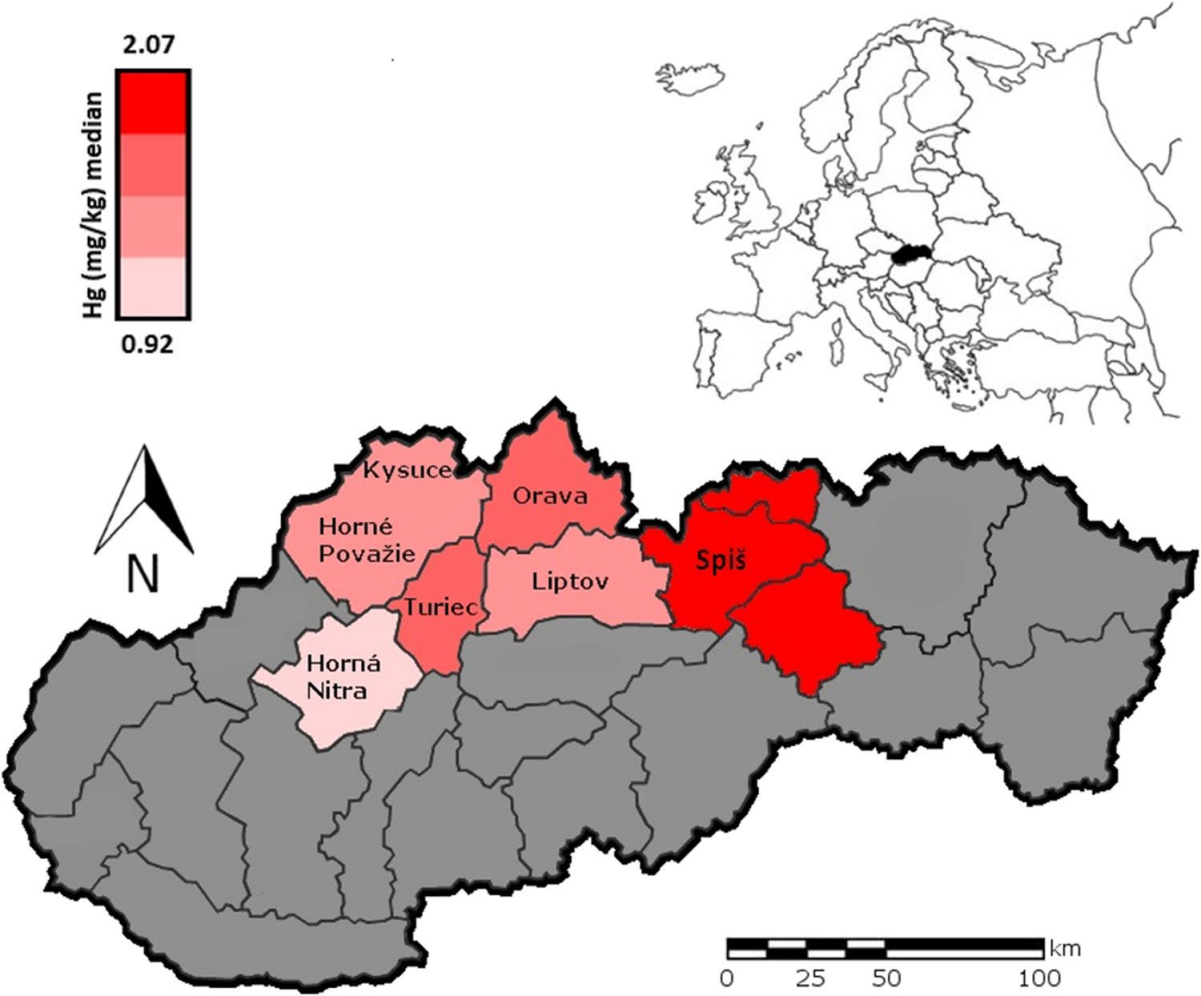
Geographical representation of the study area within Slovakia and Europe. Selected regions are highlighted on the map in color according to the intensity of mercury pollution (median value)

Slovakia, which is situated in the territory of the Western Carpathians, has a rich industrial and mining history with a strong pollutive impact on the environment. The sampled localities, located in the Western Carpathian territory, were divided into six groups, according to historical districts. Among these groups, the highest pollution values were measured in the Spiš region (Table 1, Fig. 3).

The Spiš region is located between two important emission sources of environmental pollution. The first of these is Krompachy, responsible for lead, zinc, manganese, and copper production (Vilček et al. 2012). The second is Rudnany, responsible for mercury, lead, cadmium copper, and zinc contamination (Angelovičová and Fazekašová 2014). In recent years, mining and metallurgical activities have stopped, but Hg levels in the soil from this area still exceeded permitted limits (Musilova et al. 2016). Svoboda et al. (2000) has done research comparing the Spiš region with six other areas and reports a problem with persistent pollution despite the fact that factories have been closed since 1990 (Svoboda et al. 2000). Musilova et al. (2016), whose study also focused on mercury levels in the Spiš region, confirmed these persistently elevated Hg values (Musilová et al. 2022). Heavy metal pollution does not appear only in places where long-lasting mining and metallurgy industry is located, but also in remote areas of mountains (Fig. 3).

### Habitat

The Western Carpathians, in which Slovakia is located, show higher levels of metal pollution (Ballová and Janiga 2018; Ballová et al. 2019). Several studies confirmed our assumptions that heavy metal pollution in alpine mountain ranges located in the Western Carpathians is caused by long-distance transport of air pollution from Polish and Slovakian industrial locations (Camarero et al. 2009; Janiga and Haas 2019). Gaseous forms of mercury may be transported over long distances and deposited in alpine ecosystems, where their accumulation subsequently occurs. A study by Kompiš and Ballová (2021) explains that this pollution is also caused by prevailing north-western winds. We assume role of the high mountain areas as naturally occurring barriers (Rogora et al. 2006), where pollutants are absorbed from the air.

Golden eagles were used as a bioindicator to compare heavy metal accumulation in alpine and forest areas. The measured values of mercury in the feathers of the Carpathian eagle population did not significantly differ between the eagles which hunt in forests and those which hunt also in alpine zone. Our results are a bit inconsistent with large number of studies that describe animals inhabiting higher elevations as being exposed to higher concentrations of pollutants, mainly due to deposition of pollutants on mountain ridges (Ballová and Janiga 2018; Ballová et al. 2019; Janiga et al. 2019). One possible explanation is that forests tend to trap toxic metals from the atmosphere and thus provide protection from pollutants (Kocić et al. 2014; Huang et al. 2017). Janiga and Haas (2019) observed that mercury concentrations tend to be higher in songbirds living in alpine vegetation lines than in birds living in subalpine areas. The higher mercury levels in birds of prey are usually explained by their relatively high trophic position in the food chain. For example, Durmuş (2018) focused on mercury concentrations in the feathers of wild birds. The family Accipitridae, including golden eagles, had significantly higher Hg concentrations (x̄ = 0.432 mg/kg) compared to other bird families. And yet not high enough relative to other birds of prey. The author justifies this by assuming that alpine habitats are cleaner than forest habitats.

## Conclusion

We expect variability in feather Hg concentrations among habitats due to differences in dietary preferences of forest-dwelling and alpine-dwelling eagles, as well as due to heavy metal accumulation at different altitudes. Since this hypothesis has not been confirmed, we assume that the golden eagle does not hunt strictly in alpine meadows but may also frequent lower elevations, and thus have a similar diet to forest-dwelling eagles. Since the hunting territory of one pair of golden eagles can be more than 100 m^2^ depending on the amount of food availability (Sergio et al. 2004), there is a possibility that an eagle expected to hunt only in the alpine may also frequent forest ecosystems, as well as the inverse scenario.

We drew several conclusions from the results. As the measured values in different feather types did not differ significantly, we concluded that measuring mercury in any type of dropped eagle feather appears to be a suitable method for monitoring environmental pollution. No significant differences were found between regions or different habitats. As some measured values exceeded the limit associated with health complications in eagles, mercury monitoring in raptors should be continued into the future in order to ensure protection of raptors but also for the protection of the environment in general.

## Data Availability

Data matrices will be provided by corresponding author on reasonable request.
